# Promoter methylation of MCAM, ERα and ERβ in serum of early stage prostate cancer patients

**DOI:** 10.18632/oncotarget.14873

**Published:** 2017-01-28

**Authors:** Mariana Brait, Mithu Banerjee, Leonel Maldonado, Akira Ooki, Myriam Loyo, Elisa Guida, Evgeny Izumchenko, Leslie Mangold, Elizabeth Humphreys, Eli Rosenbaum, Alan Partin, David Sidransky, Mohammad Obaidul Hoque

**Affiliations:** ^1^ Department of Otolaryngology and Head and Neck Surgery, The Johns Hopkins University School of Medicine, Baltimore, Maryland, USA; ^2^ Department of Urology, The Johns Hopkins University School of Medicine, Baltimore, Maryland, USA; ^3^ Department of Oncology, The Johns Hopkins University School of Medicine, Baltimore, Maryland, USA; ^4^ Department of Pathology, University of South Alabama Medical Center, Mobile, Alabama, USA; ^5^ Department of Urological Oncology, Davidoff Center, Beilinson Hospital, Eliahu Hakim, Ramat Aviv, Israel

**Keywords:** prostate cancer, methylation, early detection

## Abstract

**Background:**

Prostate cancer (PC) is the second most common cancer among men worldwide. Currently, the most common non-invasive approach for screening and risk assessment of PC is measuring the level of serum prostate-specific antigen (PSA). However, the sensitivity of PSA is 42.8 % and specificity is 41.1%. As a result, the serum PSA test leads to numerous unneeded biopsies. Therefore, a rigorous search for biomarkers for early detection of PC is ongoing. In this study, we aim to assess a panel of epigenetic markers in an intend to develop an early detection test for PC.

**Results:**

The sensitivity and specificity of hypermethylation of *MCAM* was 66% and 73% respectively which is an improvement from the sensitivity and specificity of PSA. Considering a combination marker panel of *MCAM, ERα* and *ERβ* increased the sensitivity to 75% and the specificity became 70% for the minimally invasive early detection test of PC.

**Materials and Methods:**

Sixteen primary matched tumor and serum were analyzed by quantitative methylation specific PCR (QMSP) to determine analytical and clinical sensitivity of the genes tested (*SSBP2, MCAM, ERα, ERβ, APC, CCND2, MGMT, GSTP1, p16* and *RARβ2*). Additionally, serum samples from eighty four cases of PC, thirty controls and seven cases diagnosed as high grade Prostatic Intraepithelial Neoplasia (HGPIN) were analyzed.

**Conclusions:**

Promoter methylation of *MCAM, ERα* and *ERβ* have a potential to be utilized as biomarker for the early detection of prostate PC as their sensitivity and specificity seem to be better than serum PSA in our cohort of samples. After robust validation in a larger prospective cohort, our findings may reduce the numbers of unwarranted prostate biopsies.

## INTRODUCTION

Prostate cancer (PC) is the most frequent cancer among men in the United States and 180,890 men are estimated to be diagnosed with PC in the USA in 2016, with 26,120 estimated deaths due to this cancer type [1]. About one man in seven will be diagnosed with PC during his lifetime [2]. Early stages of PC do not cause any specific signs or symptoms, and early detection correlates with better outcomes. Therefore, there is a thrust on developing early detection tools for PC.

The main tool for diagnosis of PC is Digital Rectal Examination (DRE) and the main tool for screening and risk assessment is serum prostate-specific antigen (sPSA). After the implementation of the sPSA test in the clinic, the detection of PC dramatically increased with a peak in the early 1990s. Although, early detection of PC substantially improved due to routine sPSA testing, there is no consensus regarding whether this test effectively reduces the risk of death from the disease [3]. In patients with sPSA values between 3 and 10 ng/mL, the sPSA test has a low specificity for PC, resulting in a high negative biopsy rate of 60% to 75% [4]. The specificity is compromised by several other non-cancer associated pathological conditions, such as infections, inflammation, acute urinary retention or benign prostatic hyperplasia (BPH), resulting in high false-positive rates. The unsatisfactory correlation between sPSA levels and disease state leads to unnecessary biopsies [5]. In addition to rising health care cost, these unnecessary biopsies increase morbidities including hematuria, hematospermia, prostatitis, and also impact the patient's psychological status. Furthermore, although sPSA-based screening has reduced PC mortality by 20%, it is associated with a high risk of over diagnosing clinically insignificant PC that would not have been diagnosed in the patient's lifetime in the absence of screening [3, 6, 7]. At present, it is difficult to predict which tumor will become potentially life-threatening and which one will not.

Imaging of the prostate also has its own shortcomings. A recent meta-analysis of studies correlating magnetic resonance imaging (MRI) and histopathology found sensitivities of 37% and 96% respectively for detecting PC, with differences due to variable definitions of cancer, exclusion of transitional zone cancers and criteria used for positive findings [8]. Intra-prostatic tumor growth is associated with increased cell membrane turnover and increased cell proliferation, which lead to altered relative concentrations of certain metabolites including creatine, choline and citrate, most specifically an increase in choline and a decrease in citrate. Magnetic resonance spectroscopy (MRSI) is a technology that increases the sensitivity and specificity of MRI by analyzing the metabolic profile. The largest study comparing MRSI to MRI found that MRSI alone had a higher sensitivity (76%) than T2-weighted MRI (67%), but a lower specificity [8]. Imaging modalities can be used for screening in bigger centers and the cost could be prohibitive, therefore, the imaging approach may not be not an ideal screening tool for all the high risk group of patients specifically in the developing countries.

Promoter methylation, one of the most studied epigenetic alterations in human malignancy, refers to the addition of a methyl group to the cytosine ring of cytosines that precede a guanosine (referred to as CpG dinucleotides) to form methyl cytosine (5-methylcytosine). CpG dinucleotides are found at an increased frequency in the promoter region of many genes, and methylation in the promoter region is frequently associated with “gene silencing” [9]. Several tumor suppressor genes (TSG) contain CpG islands in their promoters and many of them show evidence of methylation regulated silencing [10, 11]. Previous studies have shown that these epigenetic changes are an early event in carcinogenesis and are present in the precursor lesions of a variety of cancers including prostate [10, 12, 13], bladder [11, 14–16] and other organs [17]. Additionally, some cancer specific methylation events can be detected in bodily fluids including serum, saliva, urine, etc. [18–20].

In the present study, we tested a panel of ten genes (*SSBP2, MCAM, ERα, ERβ, APC, CCND2, MGMT, GSTP1, p16 and RARβ2*), previously found to be methylated in PC [12, 13, 20–26], however, most of them have not been previously tested in serum from PC patients to determine the potential of these genes as non-invasive biomarkers for PC. Using quantitative methylation specific PCR (QMSP), we aimed to identify early stage PC specific methylation markers. To this end, we first determined the analytical sensitivity of each of the markers by testing a small cohort of primary PC tissues with paired serum. And subsequently, we tested an independent set of serum samples from cases and controls to assess the clinical sensitivity of individual genes or panel of genes.

## RESULTS

### Methylation in prostate primary tumor and paired serum samples

**Figure 1:**
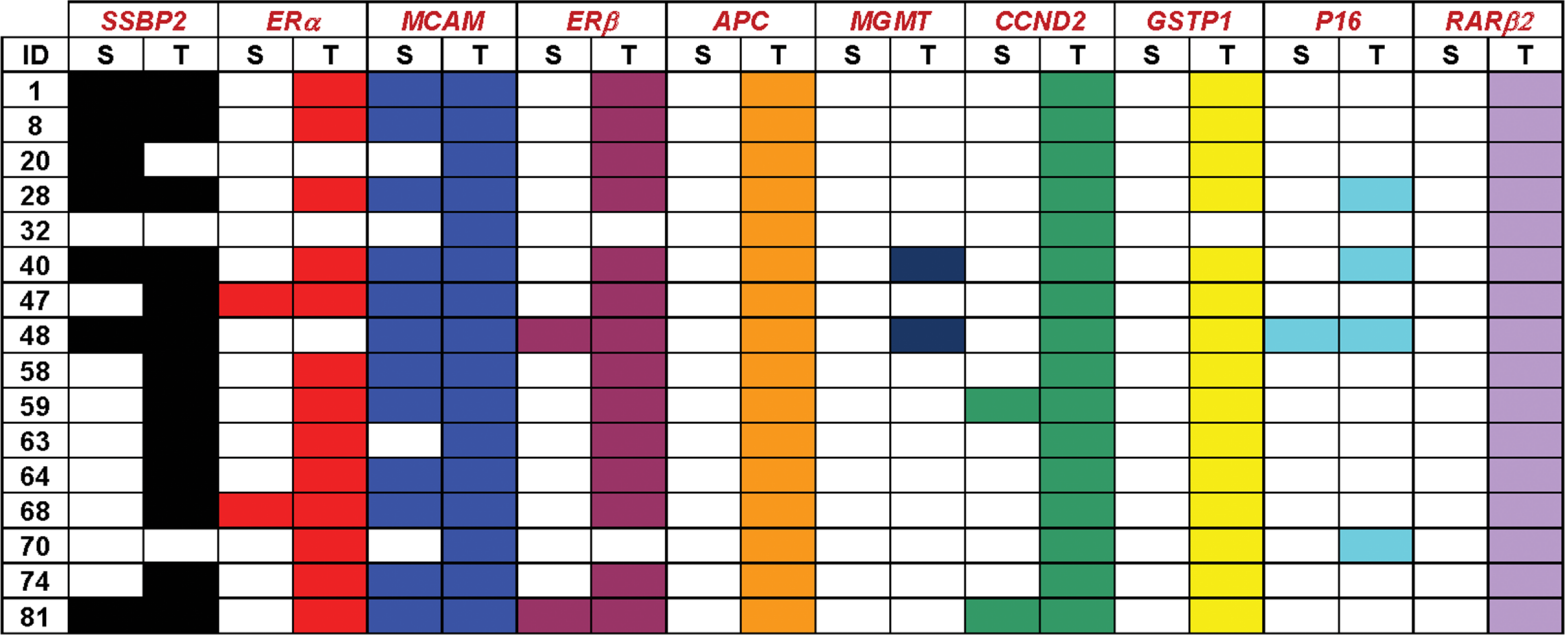
Methylation patterns of each of the ten genes in prostate primary tumor and paired serum samples Color cells represent presence of methylation in the corresponding gene in either tumor (T) or paired serum (S) samples from sixteen patients.

To determine the analytical sensitivity, we first analyzed the promoter region methylation of ten genes in paired tumor and serum samples from sixteen patients. Figure 1 summarizes the methylation profiles of each of the ten genes in tumor with paired serum samples. Each of the analyzed gene showed methylation in at least one primary tumor sample. *APC* and *RAR-*β showed methylation in all the tumor samples, however these genes did not show methylation in any of the paired serum sample. For the remaining genes (*SSBP2, MCAM, ERα, ERβ, CCND2, MGMT, GSTP1* and *p16*) methylation in serum DNA was always corresponding to methylation of tumor DNA, while methylation of tumor DNA was not always corresponding to methylation of serum DNA. No aberrant methylation (i.e., hypermethylation) was detected in the serum of PC patients who did not also have aberrant methylation of the same promoter in the corresponding tumor sample with one exception for *SSBP2*, where methylation was detected in serum but not in tumor. The methylation frequency of primary tumors and analytical sensitivity of each of the genes in serum are as depicted in Table 1.

**Table 1:** Sensitivity of the QMSP assay in serum of prostate cancer patients (16 primary prostate cancer patients and matched serum samples)

Gene	Tumors with Methylation / total number of tumors (%)	Methylation in serum/Methylation in tumor (Analytical sensitivity)
*SSBP2*	13/16 (81%)	7/13 (54%)
*ER*α	13/16 (81%)	2/13 (15%)
*MCAM*	16/16 (100%)	12/16 (75%)
*ER*β	14/16 (87.5%)	2/14 (14%)
*APC*	16/16 (100%)	0/16 (0%)
*MGMT*	2/16 (12.5%)	0/2 (0%)
*CCND2*	16/16 (100%)	2/16 (15%)
*GSTP1*	15/16 (94%)	0/15 (0%)
*P16*	4/16 (25%)	1/4 (25%)
*RAR*β2	16/16 (100%)	0/16 (0%)

### Determining the feasibility of serum DNA promoter methylation of a panel of 10 gene for the detection of PC in an independent set of cases and controls

In this set of samples, a total of 84 serum samples from cases and 37 cancer free control serum samples were tested by QMSP for all the 10 genes. The demographic and clinical characteristics of the 84 PC patients included in this study are summarized in Table 2. Briefly, the median age of cases was 59 years (range 39–76 years) and the median age of control was also 59 (range 49–71). Serum PSA level was between 2.6 ng/ml to 9.9 ng/ml for the majority of cases. We had sPSA data for 10 controls, that had a negative biopsy, and it ranged from 3.7 to 8.9 ng/mL. Among the 37 controls no known pathological conditions existed in 30 samples and the remaining 7 samples were HGPIN, which is known to be a PC precursor lesion. 20 out of the 30 were considered as cancer free controls exposed to potential cancer risk factors such as smoking, high fat diet etc.[10]. All PC cases were confirmed by standard histopathology.

**Table 2:** Demographic and clinicopathological characteristics of the 84 prostate cancer patients used in this study

Characteristic	Number of patients (%)
**Age (years)**	
Median age	59 (range 39–76)
> 59	38 (45%)
≤ 59	46 (55%)
**Race**	
Caucasian	40 (48%)
African-American	44 (52%)
**Clinical Stage**	
T1c	60 (72%)
T2a	7 (8%)
T2b	2 (2%)
T3	1 (1%)
Unknown	14 (17%)
**Gleason score**	
2–4	0 (0%)
5–6	65 (78%)
7	17 (20%)
8–10	2 (2%)
**PSA level (ng/mL)**	
0–2.5	5 (6%)
2.6–9.9	66 (79%)
10–19.9	10 (12%)
> 20	3 (3%)

**Figure 2:**
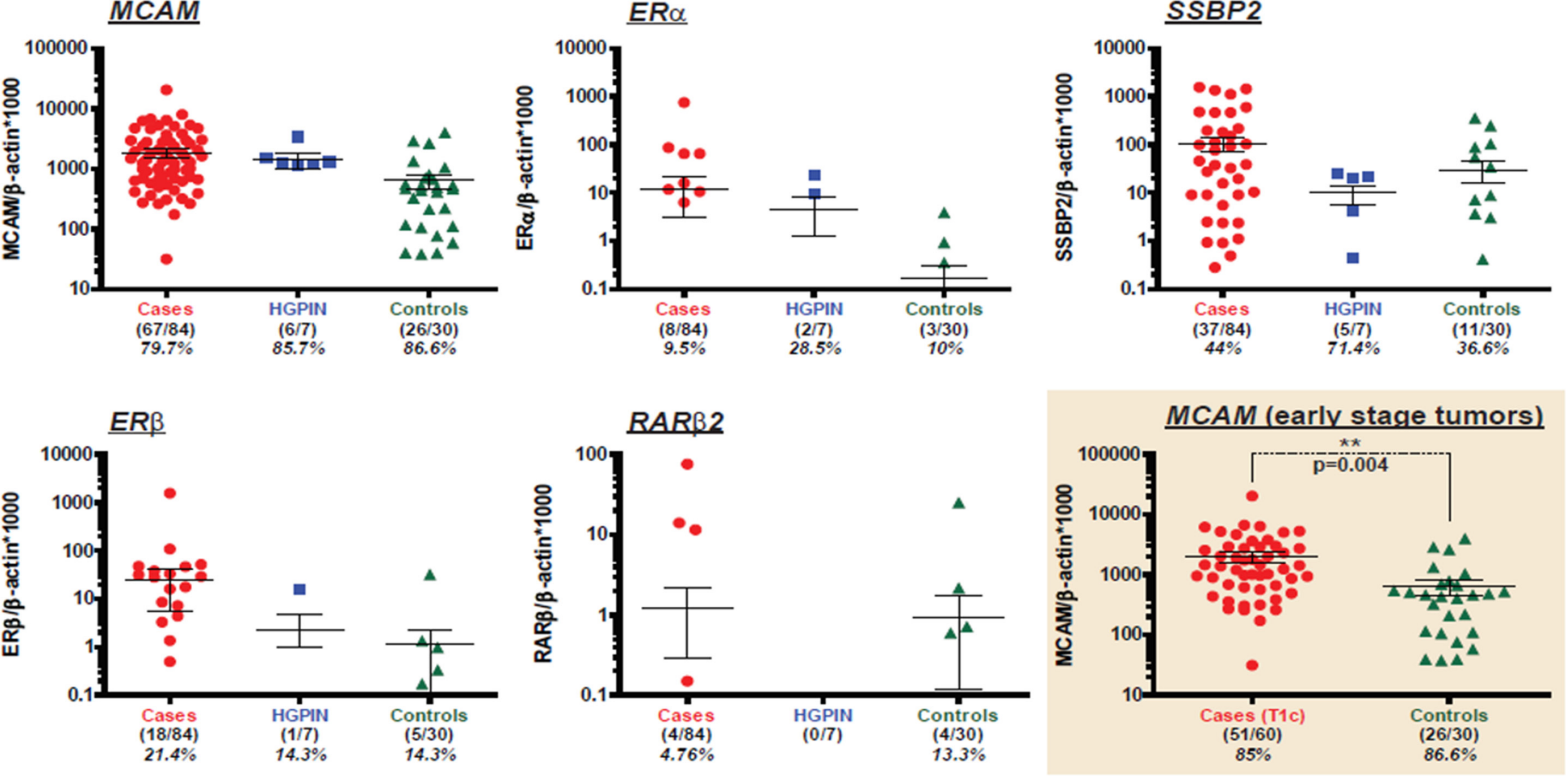
Methylation of *MCAM*, *ERα*, *SSBP2*, *ERβ* and *RARβ2* genes in cases, controls and HGPIN samples Scatter plots demonstrate methylation levels of *MCAM*, *ERα*, *SSBP2*, *ERβ* and *RARβ2* genes in serum from prostate cancer cases (red, *n* = 84), HGPIN (blue, *n* = 7) and controls (green, *n* = 30). Mean value ± SEM (error bars) are shown. Colored frame indicates *MCAM* methylation levels detected in serum of patients with early stage (T1c) tumors (red, *n* = 60) and controls (green, *n* = 30). *p* = 0.004 for early stage cases vs. controls.

We first generated ROC curves using 84 cases and 10 controls for each of the gene to determine cutoff values for all 10 genes by maximizing sensitivity and specificity (7 HGPIN samples and 20 controls exposed to potential cancer risk factors were excluded from the control group for this analysis). The ROC curves are depicted in Supplementary Figure 1. The areas under the curve (AUC), cutoff values, sensitivity and specificity of all the genes are depicted in Table 3. The methylation values of *MCAM, ER*α, SSBP2, *ER*β and *RAR*β2 genes in cases, controls and HGPIN are shown in Figure 2.

**Table 3:** The AUC, cutoff, sensitivity & specificity of genes in serum

Gene	AUC	Cutoff	sensitivity	specificity
***MCAM***	0.66	543.37	65.5%	73.3%
***SSBP2***	0.55	9.0	33.3%	80.0%
***ERα***	0.50	6.29	9.5%	100%
***RARβ2***	0.44	75.6	1.2%	100%
***ERβ***	0.52	1.37	20.2%	96.7%
***CCND2***	0.52	0.64	4.7%	100%
***GSTP1***	0.47	105.92	1.2%	100%
***P16***	0.50	50.28	1.2%	100%
***APC***	0.55	0	N/A*	N/A
***MGMT***	0.50	0	N/A	N/A
**sPSA**	0.51	29 (*optimal*)	20.0%	96.4%
**sPSA**	0.51	4 (*clinical*)	77.4%	30%

*N/A, not assessable due to low or absent values.

The table reports the parameters of the ROC curve of the 10 genes tested in 84 serum samples from PC patients and 10 control subjects (7 HGPIN samples and 20 controls exposed to potential cancer risk factors were excluded from the control group).

**Figure 3:**
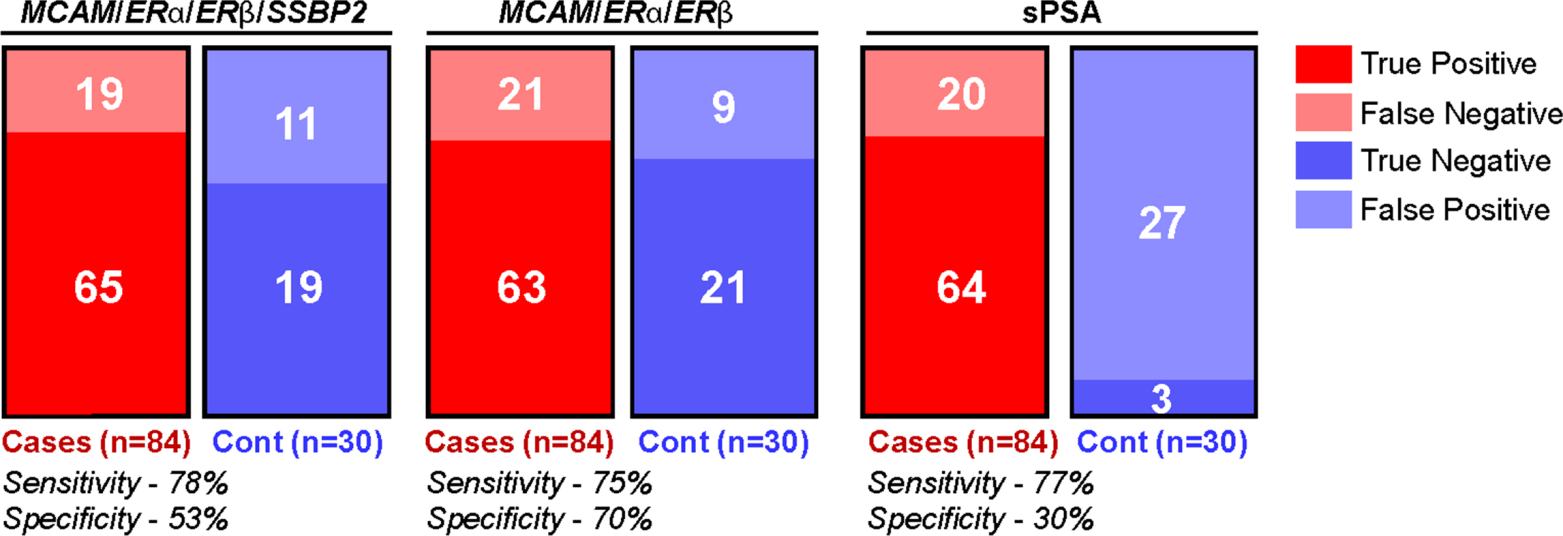
Methylation of selected genes panel demonstrate equal sensitivity and increased specificity compared to sPSA Schematic representation of true positives (red), false negatives (pale red), true negatives (blue) and false positive (pale blue) detected in serum of PC patients and controls by the panel of 4 genes (*MCAM*, *ERα*, *ERβ*, *SSBP2*), 3 genes (*MCAM*, *ERα*, *ERβ*) or sPSA. Corresponding sensitivity and specificity is shown.

Among the tested genes, *MCAM* showed the highest AUC value and the best sensitivity (Table 3 and Supplementary Figure 1). Out of a total of 84 cases, 67 cases (80%) were methylation positive for MCAM when we considered zero as the cut off. Seventy-two percent (60/84) of our serum samples were tested from patients with T1c tumors (Table 2). Fifty-one out of these 60 cases (85%) were *MCAM* methylation positive at zero cut off (Figure 2), which highlights the potential for detection of neoplastic changes at the early stage of the disease. Among the 9 T1c cases in which *MCAM* promoter methylation was not detected in the serum, sPSA levels were below 4 ng/ml in 3 of these 9 cases (data not shown). In order to maximize sensitivity and specificity, we established a cutoff of 543 for *MCAM* based on the ROC, and 40/60 (67%) of the T1c cases were positive by this cutoff value. With a cutoff of 4 ng/ml for sPSA, 15 out of the 20 missed samples could be picked up. Therefore, by combination of *MCAM* promoter methylation and sPSA level above 4 ng/ml would able to detect 55/60 (91.6%) of the T1c cases. When we considered methylation of at least one of four genes (*MCAM, ERα, ERβ* and *SSBP2)* in our tested serum samples (tumor cases and controls) similar sensitivity as of sPSA was obtained (77% for sPSA versus 78% for gene combination), however, the specificity was increased by combination gene panel (30% for sPSA versus 53% for 4 genes combination) (Figure 3). Combinations of 3 genes panel (*MCAM, ERα, ERβ*) has demonstrated similar sensitivity (75%) with even higher specificity (70%).

The mean methylation levels of the genes are depicted in Table 4. The mean methylation values for *MCAM* showed significant differences when values in cases were compared to controls (all 30 normal samples included). Although not significant, *SSBP2*, *ERα* and *ERβ*, showed a trend of higher mean methylation level in cases compared to controls. Finally, when mean methylation level of *ER*α and *MCAM* were compared in cancer cases, HGPIN and controls, there is an increase in trend from controls to HGPIN and then to cancer but it was not found to be statistically significant. Since the number of HGPIN in our study is very limited (seven), these findings are very preliminary.

**Table 4:** The table depicts mean methylation levels values (± SEM) for the 10 tested genes in serum from cancer cases (n = 84) and controls samples (n = 30)

Mean methylation levels (± SEM)			
Gene	Controls (*n* = 30)	Cases (*n* = 84)	*P* value
***SSBP2***	29.75 ± 14.28	106.0 ± 33.27	0.038
***ERα***	0.1690 ± 0.129	11.98 ± 8.969	0.192
***MCAM***	632.3 ± 168.3	1793.2 ± 299.3	0.001
***ERβ***	1.152 ± 1.055	24.29 ± 18.67	0.219
***CCND2***	0.00 ± 0.00	0.6993 ± 0.401	0.085
***GSTP1***	0.0310 ± 0.029	1.261 ± 1.261	0.332
***P16***	0.00 ± 0.00	0.5986 ± 0.486	0.320
***RARβ2***	0.9453 ± 0.828	1.21 ± 0.922	0.833
***APC***	N/A	N/A	N/A
***MGMT***	N/A	N/A	N/A

*N/A, not assessable due to low or absent values.

The *p*-values for the difference in mean methylation levels between cancer cases and controls are shown for each gene.

### Association of methylation with clinico-pathological parameters

Association of methylation and clinico-pathological variables like age, Gleason score, stage and PSA level did not show any significance. However, promoter methylation of *SSBP2* gene was shown to be more frequently methylated in Caucasian compared to African American PC cases (*p* < 0.008).

## DISCUSSION

In the present times, the cornerstone of non-invasive risk assessment/screening for the detection of PC is serum PSA. However, serum PSA has a poor sensitivity and specificity which leads to a significant number of men being subjected to unnecessary biopsies. On the other hand, there is a possibility of missing significant cancers in patients with “normal” PSA values. This study was designed to evaluate a panel of epigenetic markers for non-invasive early detection of PC that may have potential to decrease the number of unnecessary prostate biopsy.

In our study, we identified promoter methylation of *MCAM* in serum as the best performing gene with a sensitivity and specificity of 65.5% and 73.3%, respectively compared to 77.4% and 30% for PSA,. Hence, if confirmed, hypermethylation of *MCAM* in serum may have the potential to be included in a PC serum marker panel. Interestingly, 6 out of 7 serum samples from HGPIN showed methylation of *MCAM* which could indicate that these HGPIN should be followed carefully. However, a larger number of HGPIN need to be tested to determine its potential clinical utility. In our previous study, *MCAM* promoter methylation was directly correlated with tumor stage (pT3 + pT4) (*P* = 0.001) and Gleason score (*P* = 0.018) in primary prostate carcinoma tissue [12]. In the present study, where most of the cases are T1, we have not observed any statistically significant association in serum DNA methylation of *MCAM* with clinical stage of the disease that may be due to small number of sample size in each stage. To our knowledge, we are the first to explore the possibility of detecting this marker in serum.

Among other genes tested, we have previously reported 61% promoter methylation of *SSBP2* in prostate tumors [23]. Here we observed 81% promoter methylation in primary tumors. This discrepancy may be due to the small sample size in both studies. In this study, in our paired tumor-serum matched samples, one case showed methylation in serum while methylation was absent in primary tumor. This case had a PSA of 8.3 and a Gleason score of 7 (case 20, Figure 1). This serum sample may contain tumor cells from a different lesion since PC can be multifocal, and may reflect the tumor heterogeneity existing in PC [27]. Hypermethylation of *APC* was detected in 73% of PC samples in a previous study [26]. In contrast, we found 100% promoter methylation in our primary tumor samples. This could be attributed to differences in techniques as well as CpG site analyzed.

*MGMT*, a DNA repair gene showed methylation in 12.5% of our tumor samples, however we did not observe methylation of *MGMT* in any of the serum samples (initial set and independent set of cases and controls). *p16*, a modulator of the cell cycle showed 25% hypermethylation in primary PC tissues in our study. Previous studies reported *p16* hypermethylation in up to 47.6% of prostate tumor tissues with good and bad prognosis [21]. There are currently no studies where promoter methylation of MGMT and p16 was tested in the serum of PC cases. The later study also reported hypermethylation of *RAR-β*2 in PC with inconsistent prognosis. In all of our tumor tissue samples, *RAR-β*2 was methylated, however, in serum only 1.1% samples were methylated. So, *RAR-β*2 is not a promising marker for the detection of PC by serum analysis. Similarly, although the frequency of *GSTP1* promoter methylation, is high in primary PC, it is not a suitable methylation marker for the detection of PC in serum due to low sensitivity. *GSTP1* methylation has been studied in plasma and serum from PC samples and the reported frequencies range from 2 to 30% [28]. We observed a low frequency (less than 2%) of *GSTP1* methylation.

After fine-tuning the assay of *ERα*, it was included in a marker panel because it independently detects about 10% of PC in serum with high specificity. Moriyama-Gonda et al. reported hypermethylation of *ERα* in 33, 50 and 56% of PC cases with Gleason score of < 7, = 7 and > 7 respectively [25]. We determined *ERα* promoter methylation in 81% of primary tumor tissue samples. This discrepancy may be due to using a more sensitive assay by our group, whereas the later study used conventional methylation specific PCR.

When compared to studies that used similar methylation detection, we found similar methylation frequency of *ER-β* in primary PC tissue as previously reported [24]. Due to reasonably better sensitivity and specificity in serum samples, *ER-β* should be tested in a larger cohort of serum samples to determine the feasibility of its future inclusion in non-invasive serum marker panel for PC.

In a recent study, we determined methylation of some cancer associated genes in serum samples from high-risk subjects [10]. These subjects were exposed to different risk factors such as smoking, and consumption of high fat foods. Among our 30 normal controls, 20 were taken from that study, which may compromise the specificity of some of the genes tested.

In our studied serum samples, PSA has a sensitivity of 77% and specificity of 30%. When we considered promoter methylation of a 3 gene panel (*MCAM, ERβ and ERα*), the observed sensitivity and specificity was 75% and 70% respectively. After further validation, these markers can be used independently or in combination with sPSA for PC screening. Since 72% of our cases are PT1c tumors, these serum markers can be used to detect early PC that usually don't present any symptoms. This would also reduce the number of unwanted prostate biopsies and thus reduce the associated morbidity.

In summary, we have tested a panel of ten genes to identify non-invasive biomarkers with an intent to decrease biopsy for early stage PC. Since the study is done in serum, this could be utilized as a non-invasive tool for detection of PC in parallel with routine screening for PSA by a single blood draw. Future multicenter studies with larger cohorts of patients, with complete clinical and pathological information, and including different clinical stages and HGPIN should be performed to clarify the promise of these markers in clinical decision making.

## MATERIALS AND METHODS

### Study population

We evaluated serum samples from 84 PC cases and 30 cancer free controls. Additionally, we analyzed 7 high grade Prostate Intraepithelial Neoplasia (HGPIN). All PC cases had undergone prostate biopsy and were assessed for Gleason score and tumor stage. Sixteen formalin fixed paraffin embedded (FFPE) prostate tumor tissues were available from the later 84 PC cases. All patients underwent therapeutic surgery and/or biopsy at The Johns Hopkins Hospital. The serum samples were obtained from the Urology Department's bio-repository (headed by Dr. Alan Partin). The demographic and clinical information was obtained from the computerized tumor registry at The Johns Hopkins Healthcare System. A detailed summary of the demographic and clinico-pathological parameters for all the PC patients is available in Table 2. In this retrospective pilot study, some clinical information of few patients are missing in our clinical database. However, we performed all the calculation based on those samples with available clinical parameters. Approval for research on human subjects was obtained from The Johns Hopkins University institutional review boards. Samples were de-identified by study number. So this study qualified for exemption under the U.S. Department of Health and Human Services policy for protection of human subjects [45 CFR 46.101(b)].

### Gene selection

We have selected and analyzed promoter methylation of 10 genes (*SSBP2, MCAM, ERα, ERβ, APC, CCND2, MGMT, GSTP1, p16 and RARβ2*), that have been previously reported in PC primary tissue by our group and others [12, 13, 20–26].

### DNA extraction

After obtaining the informed consent, a blood sample was collected in a 10 ml vacutainer tube. Following centrifugation at 1000 × g for 10 minutes at 4°C, the separated serum was stored at –80^°^C. DNA from serum was extracted as previously described [18, 29]. Briefly, DNA was obtained from 1 mL of serum by digestion with 50 μg/mL proteinase K (Boehringer Mannheim, Germany) in the presence of 1% sodium dodecyl sulfate (SDS) at 48°C for 3 days, followed by phenol/chloroform extraction and ethanol precipitation and finally dissolved in 20 μL of LoTE (2.5 mmol/L EDTA and 10 mmol/LTris-HCL) and stored at –20^°^C until used. DNA from primary tumor tissues was extracted as described previously [19].

### Bisulfite treatment and QMSP

DNA was treated with sodium bisulfite, which converts unmethylated cytosine residues to uracil residues, using EpiTect Bisulfite kit (Cat No. 59104, from QIAGEN Inc. Valencia, CA), following the manufacturer's instructions. Bisulfite-converted DNA was used as a template for fluorescence-based real-time PCR. Amplification reactions were carried out in triplicate in a final volume of 20 μL that contained 2 μL of bisulfite-modified DNA; 600 nM concentrations of forward and reverse primers; 200 nM probe; 0.6 U of platinum Taq polymerase (Invitrogen, Frederick, MD); 200 μM concentrations each of dATP, dCTP, dGTP and dTTP; and 6.7 mM MgCl_2_. Primers and probes were designed to specifically amplify the promoter region of SSBP2, *ER*α, *MCAM*, *ER*β, *MGMT*, *APC*, *CCND2*, *GSTP1*, *p16*, *RAR*β2, and of a reference gene, β*-actin*; primer and probe sequences are provided in Supplementary Table 1. Amplifications were carried out in 384-well plates in a 7900HT sequence detector (Applied Biosystems, Foster City, CA) using the following conditions: 95°C for 3 minutes, followed by 50 cycles at 95°C for 15 seconds and 60°C for 1 minute. Results were analyzed by a sequence detector system (SDS 2.4; Applied Biosystems). Each plate included patient DNA samples, positive and negative controls. Serial dilutions (90–0.009 ng) of *in vitro* methylated DNA were used to construct a calibration curve for each plate. The relative level of methylated DNA for each gene in each sample was determined as a ratio of methylation specific PCR-amplified gene to β*-actin* (reference gene) and then multiplied by 1000 for easier tabulation (average value of triplicates of gene of interest divided by the average value of triplicates of β*-actin* × 1000).

### Statistical analysis

The optimal cutoff of methylation value for distinguishing between tumor and normal (maximizing sensitivity and specificity) was calculated using receiver-operating characteristic (ROC) curves for each gene as well as for sPSA. This was done by sorting out the methylation scores for each gene and checking the sensitivity and specificity in each unique score. To assess whether methylation levels of each gene are normally distributed, the Shapiro-Wilk test was performed, which showed that methylation values did not follow a normal distribution. Therefore, the difference in methylation levels of each gene in controls, PC cases and HGPIN were compared using non-parametric tests; the Mann-Whitney *U* test to compare differences between two groups and the Kruskall-Wallis test to compare differences among three groups. Tests for association between clinico-pathological features like age, race, stage, Gleasons score, sPSA value and methylation of each gene were performed using the Fisher's exact test and the Mann-Whitney *U* test were used for categorical and continuous variables, respectively. For continuous variables, data are expressed as mean+standard error of the mean (S.E.M). *P <* 0.05 was considered to indicate statistical significance. All statistical analyses were conducted using JMP 12 software package (SAS Institute, Cary, NC, USA).

## SUPPLEMENTARY MATERIALS FIGURES AND TABLES


