# Supplementary material for: Promoter methylation of MCAM, ERα and ERβ in serum of early stage prostate cancer patients
Source: Oncotarget. 2017 Jan 28;8(9):15431–40. doi: 10.18632/oncotarget.14873 (PMC5362497; doi:10.18632/oncotarget.14873)
Supplement: Supplementary file 1 [file oncotarget-08-15431-s001.pdf]

Promoter methylation of *MCAM, ERα* and *ERβ* in serum of early stage prostate cancer patients

Supplementary Materials

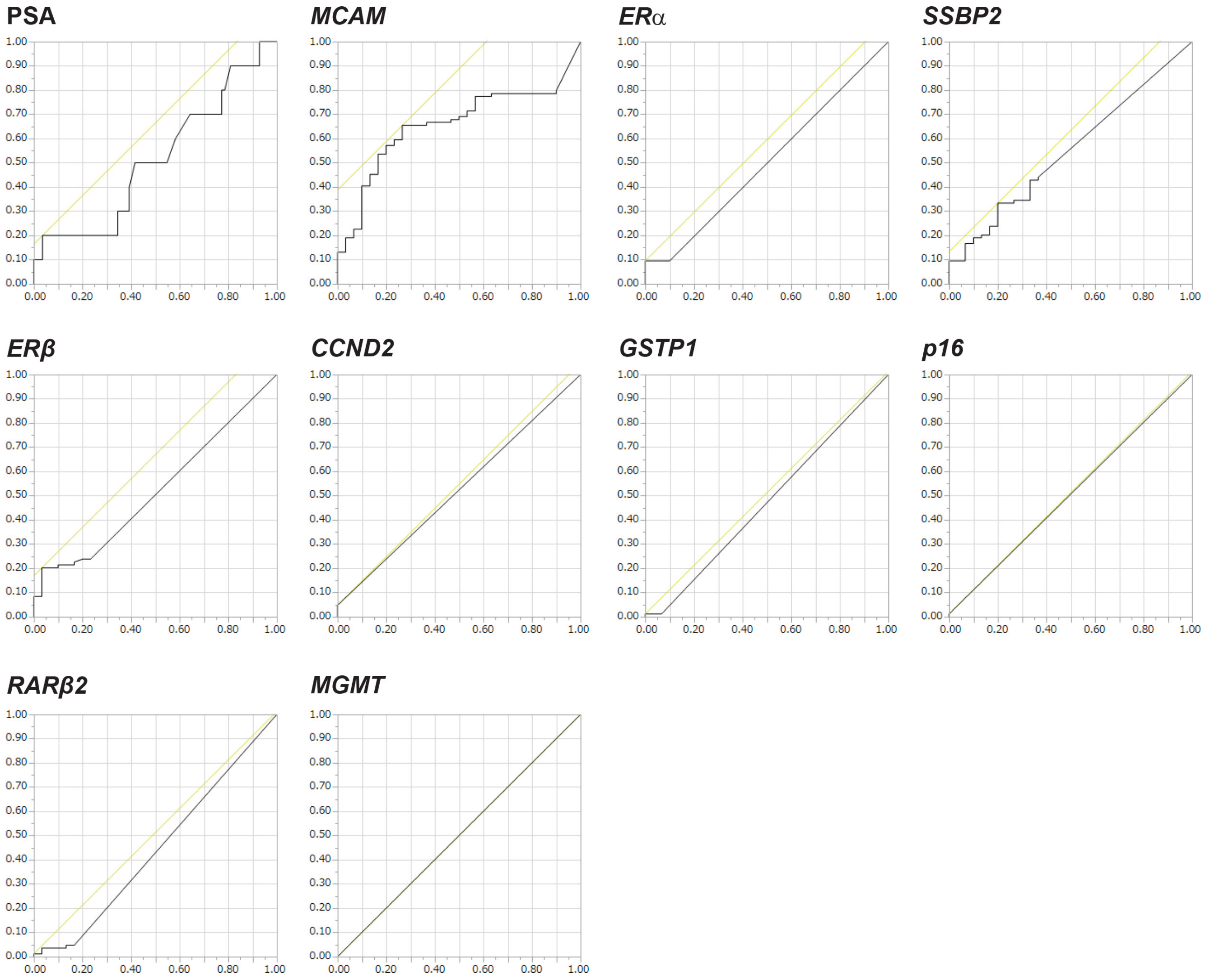

Supplementary Figure 1: Receiver Operating Characteristic (ROC) curves of each tested gene methylated in serum form 84 cancer patients and 10 control subjects (7 HGPIN samples and 20 controls exposed to potential cancer risk factors were excluded from the control group).

Supplementary Table 1: Table reports primers and probe sequences designed to specifically amplify the promoter region of *SSBP2*, *ERα*, *MCAM*, *ERβ*, *MGMT*, *APC*, *CCND2*, *GSTP1*, *p16*, *RAR*b2 and of a reference gene, β-actin

Gene	Forward Primer sequence 5′–3′	Probe sequence 6FAM 5′–3′TAMRA	Reverse Primer sequence 5′–3′
SSBP2	ATTTTTGCGGTCGTAGCGGT	ATATCCAAAACGCCGCGAAACTCC	TTCTACGACAAACTCTAACGAA
ERα	TAGGGAGTAG	CGATAAAACCGAACGACCCGACGA	GCCGACACGCGAACTCTAA
MCAM	AGAATTTAGGTCGGTTTTTATCG	ACAATATCAAACCGACGACAACGAC	ACGCAAAATTCTTCTCCCAAAA
ERβ	GGCGTTCGTTTTGGGATTG	CGATAAAACCGAACGACCCGACGA	GCCGACACGCGAACTCTAA
APC	GAACCAAACGCTCCCCAT	CCCGTCGAAAACCCGCCGATTA	TTATATGTCGGTTACGTGCGTTTA
MGMT	CGAATATACTAAAACAACCCGCG	AATCCTCGCGATACGCACCGTTTACG	GTATTTTTTCGGGAGCGAGGC
CCND2	TTTGATTTAAGGATGCGTTAGAGTACG	AATCCGCCAACACGATCGACCCTA	ACTTTCTCCCTAAAAACCGACTACG
GSTP1	AGTTGCGCGGCGATT	CGGTCGACGTTCGGGGTGTAGCG	GCCCCAATACTAAATCACGACG
P16	TTATTAGAGGGTGGGGCGGATCGC	AGT AGTATGGAGTCGGCGGCGGG	GACCCCGAACCGCGACCGTAA
RARβ2	GGGATTAGAATTTTTTATGCGAGTTGT	TGTCGAGAACGCGAGCGATTCG	TACCCCGACGATACCCAAAC
β-Actin	TGGTGATGGAGGAGGTTTAGTAAGT	ACCACCACCCAACACACAATAACAAACACA	AACCAATAAAACCTACTCCTCCCTTAA
